# Serum Glial Fibrillary Acidic Protein Can Predict Cross-Sectional Vasculitis Activity by Reflecting Renal Involvement in Patients with Antineutrophil Cytoplasmic Antibody-Associated Vasculitis

**DOI:** 10.3390/medicina60101639

**Published:** 2024-10-07

**Authors:** Lucy Eunju Lee, Taejun Yoon, Jihye Chung, Jang Woo Ha, Yong-Beom Park, Sang-Won Lee

**Affiliations:** 1 Division of Rheumatology, Department of Internal Medicine, Yonsei University College of Medicine, Seoul 03722, Republic of Korea; elee@yuhs.ac (L.E.L.); jchung0831@yuhs.ac (J.C.); hjwnmk@yuhs.ac (J.W.H.); yongbpark@yuhs.ac (Y.-B.P.); 2 Department of Medical Science, BK21 Plus Project, Yonsei University College of Medicine, Seoul 03722, Republic of Korea; tjyoonn92@gmail.com; 3 Institute for Immunology and Immunological Diseases, Yonsei University College of Medicine, Seoul 03722, Republic of Korea

**Keywords:** glial fibrillary acidic protein, estimate, activity, antineutrophil cytoplasmic antibody, vasculitis

## Abstract

*Background and Objectives:* Glial fibrillary acidic protein (GFAP) is a type III intermediate filament protein primarily produced by cells in the central nervous system (CNS) and other major organs such as the kidneys. This study investigated whether serum GFAP could be used to estimate cross-sectional vasculitis activity presented via the Birmingham vasculitis activity score (BVAS) in patients with antineutrophil cytoplasmic antibody-associated vasculitis (AAV). *Materials and Methods:* This study included 74 patients with AAV. Clinical and laboratory data at diagnosis including BVAS and C-reactive protein (CRP) were reviewed. During follow-up, all-cause mortality and end-stage kidney disease (ESKD) were considered poor outcomes. Serum GFAP was measured from sera collected and stored at diagnosis. *Results:* The median age of the 74 patients was 63.5 years. Serum GFAP was inversely correlated with the cross-sectional BVAS (r = −0.373) and CRP (r = −0.320). It was also significantly correlated with general (r = −0.237) and renal (r = −0.335) manifestations among BVAS systemic items, and furthermore, among minor items of renal manifestation, correlating with sum scores for proteinuria (r = −0.409) and haematuria (r = −0.305). Additionally, compared with patients with serum GFAP > 194.9 pg/mL, those with serum GFAP ≤ 194.9 pg/mL showed a higher risk for progression to ESKD (relative risk 3.150) and a significantly lower cumulative ESKD-free survival rate. *Conclusions:* This study demonstrated the clinical potential of serum GFAP at diagnosis for predicting not only cross-sectional vasculitis activity through the anticipation of the extent of renal involvement but also future progression to ESKD in patients with AAV.

## 1. Introduction

Antineutrophil cytoplasmic antibody (ANCA)-associated vasculitis (AAV) is a form of small-vessel vasculitis characterized by fibrinoid necrotizing inflammation, with minimal or absent immune deposits primarily affecting capillaries, venules, arterioles, and occasionally, medium-sized arteries [1,2]. AAV is further classified into three subtypes based on clinical features and the type of ANCA present: microscopic polyangiitis (MPA), granulomatosis with polyangiitis (GPA), and eosinophilic granulomatosis with polyangiitis (EGPA) [3,4,5].

In clinical settings, the Birmingham Vasculitis Activity Score (BVAS) is the most widely used tool for evaluating disease severity at a specific point in time. BVAS assesses disease activity across nine systemic domains, with a primary focus on major organ systems, allowing for a comprehensive and longitudinal assessment of the patient’s overall status [6]. However, BVAS has its limitations. It can be time-consuming to complete and relies heavily on the subjective judgment of individual assessors. These drawbacks highlight the need for more convenient and objective biomarkers that can accurately reflect vasculitis activity and inflammation, such as those measured by BVAS, erythrocyte sedimentation rate (ESR), and C-reactive protein (CRP) [7].

Glial fibrillary acidic protein (GFAP), a type III intermediate filament protein primarily produced by astrocytes in the central nervous system (CNS), plays a critical role in maintaining the structural integrity of the cytoskeleton [8]. Serum GFAP levels have been shown to correlate with disease activity or tissue damage in various neurological disorders involving the brain and spinal cord [9,10]. Interestingly, GFAP has also been detected in non-neurological tissues such as the kidneys, skin, and liver [11,12], suggesting that serum GFAP could potentially serve as a biomarker for AAV by reflecting inflammation in these affected organs.

The potential of GFAP as a biomarker extends beyond its well-established role in neurological conditions. Given its presence in both CNS and non-CNS tissues, GFAP shows promise for monitoring systemic inflammation in diseases like AAV [13]. Since AAV can affect multiple organ systems, including GFAP-expressing tissues such as the kidneys and liver, serum GFAP levels may offer valuable insights into the extent of systemic inflammation. Detecting GFAP in peripheral blood not only reflects CNS-related damage but also provides a broader perspective on disease activity in other affected organs. Establishing GFAP as a reliable biomarker could improve clinical assessments by offering a more accessible, reproducible, and objective measure, potentially leading to earlier detection of disease flares and more accurate monitoring of treatment response.

In the context of kidney disease, GFAP may serve as an important marker of renal inflammation or damage. Several studies have reported altered GFAP levels in the serum of patients with systemic conditions that affect the kidneys [14]. Given that the kidneys are a common target in AAV, tracking serum GFAP levels could provide critical insights into both disease activity and the extent of renal involvement. Additionally, due to GFAP’s potential role in maintaining cellular integrity and responding to inflammation [15], it could serve as an indicator of the progression or severity of kidney damage in inflammatory diseases.

To date, no studies have specifically examined the utility of serum GFAP in estimating disease activity in AAV patients as measured by BVAS. Therefore, this study aims to explore whether serum GFAP can serve as a reliable biomarker for assessing cross-sectional vasculitis activity in AAV patients, with the goal of enhancing both objectivity and convenience in clinical evaluations.

## 2. Materials and Methods

### 2.1. Patients

We randomly selected 80 patients with AAV from the Severance Hospital ANCA-associated Vasculitides (SHAVE) cohort, a prospective observational cohort of Korean patients with AAV. Patients were screened based on the following inclusion criteria: (i) diagnosed with AAV for the first time at this tertiary hospital; (ii) diagnosed according to the revised 2012 International Chapel Hill Consensus Conference Nomenclature of Vasculitides and the 2007 European Medicine Agency Algorithm for AAV; (iii) classified as having AAV according to the 2022 American College of Rheumatology/European Alliance of Associations for Rheumatology classification criteria for MPA, GPA, and EGPA; (iv) having clear and sufficient medical records for collecting clinical data at diagnosis and during follow-up; (v) having laboratory results including at least ANCA, ESR, CRP, complete blood cell count, blood urea nitrogen, serum creatinine, serum total protein, and serum albumin at diagnosis; (vi) with serum collected and stored at diagnosis and available for use in this study; (vii) having been followed up for at least 6 months or more; (viii) no serious medical conditions mimicking AAV, such as malignancies or infectious diseases requiring hospitalization; and (ix) not having been exposed to conditions or medications known to cause false-positive ANCA results before diagnosis.

Of the 80 patients, 4 were excluded due to insufficient serum for GFAP measurement, and 2 were excluded because they simultaneously fulfilled the 2022 ACR/EULAR classification criteria for both MPA and GPA. Ultimately, 74 patients were included in this study. The study was approved by the Institutional Review Board (IRB) of Severance Hospital, Republic of Korea (IRB number 4-2016-0901). All patients provided written informed consent upon enrolment in the SHAVE cohort at the time of AAV diagnosis and blood sampling. The IRB waived the requirement for additional informed consent since it had already been obtained upon cohort entry.

### 2.2. Clinical Data

At AAV diagnosis, demographic data, including age and sex, were collected. Additionally, clinical details such as AAV subtype, ANCA type and positivity, and AAV-specific indices—including the Birmingham Vasculitis Activity Score (BVAS), the Five-Factor Score (FFS) [16], the 36-item short form survey (SF-36) [17] with its physical component summary (PCS) and mental component summary (MCS), and the vasculitis damage index (VDI) [18]—were reviewed. Comorbidities such as type 2 diabetes mellitus, hypertension, and dyslipidemia were documented. Routine laboratory test results, including ESR and CRP, were also collected.

During follow-up, poor outcomes were defined as all-cause mortality and progression to end-stage kidney disease (ESKD). The frequency of immunosuppressive drug administration was also recorded.

### 2.3. Measurement of Serum GFAP

With written informed consent, whole blood samples were collected from AAV patients. Sera were promptly isolated by allowing the blood to clot, followed by centrifugation to collect the supernatant, which was then stored at −80 °C. Serum GFAP levels were measured using an enzyme-linked immunosorbent assay (ELISA) kit (Invitrogen, Carlsbad, CA, USA) according to the manufacturer’s instructions. Briefly, standards or pretreated samples were added to ELISA wells, incubated with biotinylated detection antibody and horseradish peroxidase conjugate, followed by washes. Substrate was then added, and after 15 min, the reaction was stopped. Absorbance was measured at 450 nm, and a standard curve was generated to calculate sample concentrations.

### 2.4. Statistical Analyses

All statistical analyses were performed using SPSS version 26 (IBM Corporation, Armonk, NY, USA) for Windows (Microsoft Corporation, Redmond, WA, USA) or using R version 4.3.0. Continuous variables were expressed as medians with interquartile ranges (25th–75th percentiles), while categorical variables were presented as frequencies and percentages. Pearson’s correlation coefficient (r) was used to assess the strength and direction of linear relationships between two continuous variables.

Receiver operating characteristic (ROC) curve analysis was conducted to evaluate the diagnostic performance of continuous variables, with the area under the curve (AUC) serving as a measure of the test’s accuracy. The optimal cut-off point was determined using the ROC curve, selecting the value that maximized the sum of sensitivity and specificity. The relative risk (RR) associated with the cut-off for predicting poor outcomes was analyzed through contingency tables and assessed using the chi-square test.

Survival rates between groups were compared using Kaplan–Meier survival analysis, and differences were tested with the log-rank test. Statistical significance was defined as a *p*-value of less than 0.05 (*p* < 0.05).

## 3. Results

### 3.1. Characteristics

At diagnosis, the median age of the 74 patients was 63.5 years, with 32 males (43.2%) and 42 females (56.8%). The cohort included 35 patients (47.3%) diagnosed with MPA, 23 (31.1%) with GPA, and 16 (21.6%) with EGPA. Myeloperoxidase (MPO)-ANCA (or perinuclear (P)-ANCA) was positive in 40 patients (54.1%), while proteinase 3 (PR3)-ANCA (or cytoplasmic (C)-ANCA) was positive in 12 patients (16.2%). The median BVAS score was 5.0, with median ESR and CRP levels of 23.0 mm/h and 3.8 mg/L, respectively. Additionally, the median serum GFAP level was 259.4 pg/mL. Other laboratory results are presented in Table 1.

During follow-up, six patients (8.1%) died over a median duration of 27.1 months, and 17 patients (23.0%) progressed to end-stage kidney disease (ESKD) over a median follow-up of 26.6 months. Glucocorticoids were administered to 73 patients (98.6%), with cyclophosphamide being the most commonly used immunosuppressive drug (63.5%), followed by azathioprine (60.8%) and mycophenolate mofetil (25.7%) (Table 1).

### 3.2. Correlation Analysis of Serum GFAP with Continuous Variables at Diagnosis

**Figure 1:**
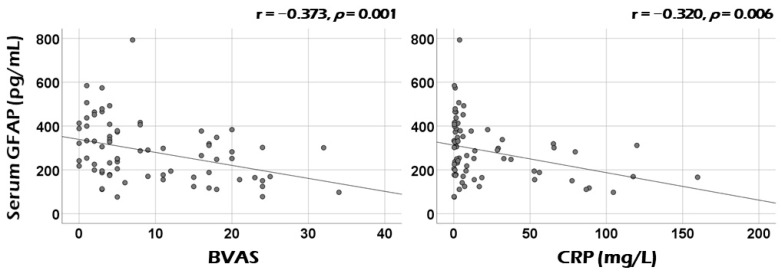
Correlation analysis: activity and acute-phase reactants. Serum GFAP was significantly correlated with cross-sectional BVAS and CRP. GFAP: glial fibrillary acidic protein; BVAS: Birmingham Vasculitis Activity Score; CRP: C-reactive protein.

Among continuous variables at diagnosis, serum GFAP was significantly correlated with cross-sectional BVAS (r = −0.373, *p* = 0.001) and CRP (r = −0.320, *p* = 0.006) (Figure 1). Additionally, serum GFAP also exhibited significant correlations with haemoglobin (r = 0.229, *p* = 0.049), and serum albumin (r = 0.232, *p* = 0.049). Serum GFAP tended to be positively correlated with age (r = 0.222, *p* = 0.057) and inversely with ESR (r = −0.209, *p* = 0.090), but these correlations were not statistically significant (Table S1).

### 3.3. Correlation Analysis of Serum GFAP with the Sum of Scores of Each Systemic Item of BVAS at Diagnosis

**Figure 2:**
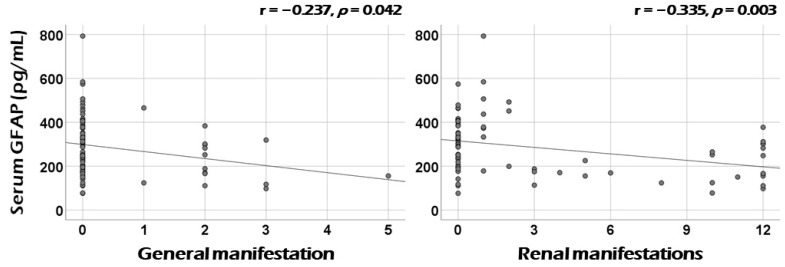
Correlation analysis: BVAS systemic items. Serum GFAP was significantly correlated with cross-sectional general and renal manifestation among the nine BVAS systemic items. GFAP: glial fibrillary acidic protein; BVAS: Birmingham Vasculitis Activity Score.

Among the nine systemic items of BVAS at diagnosis, serum GFAP was significantly correlated with cross-sectional general manifestation (r = −0.237, *p* = 0.042) and renal manifestation (r = −0.335, *p* = 0.003) (Figure 2). Additionally, otorhinolaryngologic and nervous systemic manifestations showed correlations with serum GFAP, but these did not reach statistical significance (r = −0.218, *p* = 0.062, and r = −0.218, *p* = 0.063, respectively) (Table S2).

### 3.4. Association between Serum GFAP and Renal Manifestation at Diagnosis

**Figure 3:**
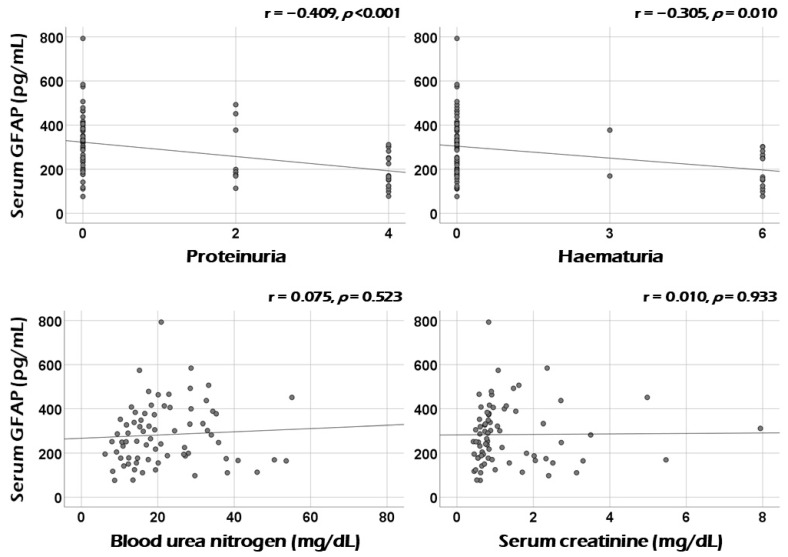
Correlation analysis: minor items of renal manifestation. Serum GFAP was significantly correlated with the sum scores of proteinuria and haematuria. GFAP: glial fibrillary acidic protein.

Among minor items of renal manifestation at diagnosis, serum GFAP was significantly correlated with the sum scores of proteinuria (r = −0.409, *p* < 0.001) and haematuria (r = −0.305, *p* = 0.010). However, serum GFAP was not correlated with blood urea nitrogen (r = 0.075), serum creatinine (r = 0.010), or creatinine clearance (r = −0.14). (Figure 3 and Supplementary Figure S2).

### 3.5. Relative Risk of Serum GFAP for Progression to ESKD

**Figure 4:**
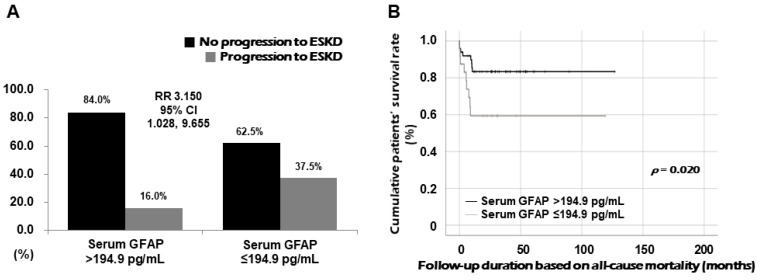
Correlation RR for ESKD and comparison of ESKD-free survival rates. Compared with patients with serum GFAP > 194.9 pg/mL, those with serum GFAP ≤ 194.9 pg/mL showed a higher risk for progression to ESKD (**A**) and a significantly lower cumulative ESKD-free survival rate (**B**). RR: relative risk; ESKD: end-stage kidney disease; GFAP: glial fibrillary acidic protein.

Using ROC curve analysis, which showed an inverse association, the cut-off value of serum GFAP at diagnosis for progression to ESKD during follow-up was determined as 194.9 pg/mL (sensitivity, 73.7%; specificity, 52.9%). When patients were divided into two groups based on serum GFAP ≤ 194.9 pg/mL at diagnosis, progression to ESKD was identified more frequently in patients with serum GFAP ≤ 194.9 pg/mL than those with serum GFAP > 194.9 pg/mL (37.5% vs. 16.0%, *p* = 0.040). Furthermore, patients with serum GFAP ≤ 194.9 pg/mL at diagnosis exhibited a significantly higher risk for progression to ESKD during follow-up than those with serum GFAP > 194.9 pg/mL (RR 3.150, 95% confidence interval [CI] 1.028, 9.655) (Figure 4A). However, we failed to demonstrate the predictive potential of serum GFAP at diagnosis for all-cause mortality.

To evaluate the independent contribution of serum GFAP ≤ 194.9 pg/mL at diagnosis to progression to ESKD during follow-up in patients with AAV, we performed Cox proportional hazards analyses. In the univariable Cox analysis, serum GFAP ≤ 194.9 pg/mL at diagnosis was significantly associated with progression to ESKD (HR 2.943, 95% CI 1.130, 7.665). Additionally, ESR (HR 1.016, *p* = 0.007) and CRP (HR 1.013, *p* = 0.017) at diagnosis were significantly associated with progression to ESKD. BVAS (HR 1.048, *p* = 0.065) and serum creatinine (HR 1.254, *p* = 0.071) at diagnosis tended to be associated with progression to ESKD, but the differences were not statistically significant. In the multivariable Cox analysis including serum GFAP ≤ 194.9 pg/mL, ESR, CRP, BVAS, and serum creatinine, only ESR (HR 1.018, 95% CI 1.001, 1.035) and serum creatinine (HR 1.762, 95% CI 1.071, 2.898) at diagnosis were independently associated with progression to ESKD. Meanwhile, serum GFAP ≤ 194.9 pg/mL (HR 3.208, *p* = 0.084) tended to be independently associated with ESKD, though this did not reach statistical significance (Table S3).

### 3.6. Comparison of Cumulative Survival Rates

Patients with serum GFAP ≤ 194.9 pg/mL exhibited a significantly reduced cumulative ESKD-free survival rate compared with those with serum GFAP > 194.9 pg/mL (*p* = 0.020) (Figure 4B).

## 4. Discussion

In this study, we explored whether serum GFAP could be used to estimate cross-sectional vasculitis activity as measured by BVAS in patients with AAV, and we obtained several noteworthy findings. First, serum GFAP suitable for use to estimate cross-sectional BVAS and CRP, along with hemoglobin and serum albumin at diagnosis. Second, it reflected renal manifestations, particularly proteinuria and hematuria, as well as general manifestations. Third, patients with serum GFAP ≤ 194.9 pg/mL at diagnosis had a threefold higher relative risk of progressing to ESKD during follow-up, despite no correlation between serum GFAP and creatinine at diagnosis. Fourth, patients with serum GFAP ≤ 194.9 pg/mL had a higher frequency of progression to ESKD compared with those with higher GFAP levels. These findings suggest that serum GFAP at diagnosis can be used to estimate cross-sectional vasculitis activity and may partially predict progression to ESKD in patients with AAV.

Previous studies have shown that serum GFAP is elevated in patients with brain or spinal cord diseases compared with healthy controls [8,9,13] and is positively correlated with IL-6, a marker of inflammation, in patients with hepatic encephalopathy [19]. These studies indicate that GFAP correlates with CNS diseases and inflammation. In SLE, blood GFAP levels were shown to be elevated in patients with active neuropsychiatric manifestations [20]. Thus, we initially expected a positive correlation between serum GFAP, BVAS, and CRP, given their roles in AAV activity and inflammation. However, our study revealed a negative correlation, which contradicted our prior assumptions. To explain these discrepancies, we focused on the specific microenvironmental interactions between GFAP and the kidneys. Even though there is no strong evidence that GFAP correlates with kidney parameters or disease activity in autoimmune diseases with renal involvement, GFAP’s presence in non-CNS tissues, including the kidneys, suggests it could reflect systemic inflammation affecting the kidneys, as seen in conditions like AAV.

Our findings showed an inverse correlation between serum GFAP and renal manifestations of BVAS, particularly proteinuria and hematuria. GFAP has been reported to be expressed in glomerular mesangial cells and podocytes and is known to play a protective role in the kidneys [12]. We hypothesize that in healthy kidneys, GFAP production is normal, but in persistently inflamed or damaged kidneys, GFAP production may decrease. In such conditions, CRP levels may increase, and proteinuria and hematuria may also be present. The lack of significant correlations between GFAP and involvement scores in other major organs may support this hypothesis.

Serum GFAP was also significantly correlated with cross-sectional levels of hemoglobin and serum albumin at diagnosis. Hemoglobin and albumin, though influenced by factors like anemia and nutritional status, also reflect the degree of inflammation in AAV patients [21]. Albumin reduction in cases of glomerulonephritis due to AAV involvement should be considered an indirect marker of inflammation and protein loss from persistent proteinuria [22]. Among the variables we analyzed, significant correlations were observed, except for between serum GFAP and serum creatinine (Figure S1). This suggests that serum GFAP may have an indirect correlation with kidney function, contributing to its ability to predict progression to ESKD.

To evaluate the independent contribution of serum GFAP, we determined a cut-off value. Although the normal range of serum GFAP is generally considered to be 10–130 pg/mL, values can vary based on age, population, and diagnostic methods. We set the cut-off at 194.9 pg/mL, based on ROC curve analysis, to maximize sensitivity and specificity. Cox proportional hazards analysis showed that GFAP ≤ 194.9 pg/mL was significantly associated with ESKD progression, alongside ESR and CRP at diagnosis. Though GFAP’s association with ESKD was not statistically significant in the multivariable analysis, it still showed a tendency toward predicting ESKD progression.

Before concluding, we offer two additional insights. First, serum GFAP does not appear to predict CNS involvement in AAV as it does in brain and spinal cord diseases. In this study, GFAP tended to be inversely correlated with nervous system manifestations in AAV, though this association was weak and disappeared when certain minor items, such as peripheral neuropathy and mononeuritis multiplex, were excluded. Second, GFAP may help recognize early and subtle kidney alterations in AAV patients. Though previous studies have not strongly linked GFAP to kidney damage in inflammatory diseases, our study found that GFAP, but not serum creatinine, significantly correlated with proteinuria, a marker of early kidney damage resulting from distinct pathological changes such as a reduced fraction of normal glomeruli in ANCA-associated glomerulonephritis.

The strength of this study lies in demonstrating, for the first time, that serum GFAP can estimate cross-sectional BVAS and reflect early kidney involvement in AAV. However, this study has limitations, including the lack of sufficient kidney histological data, a small sample size, and a retrospective study design. Additionally, the absence of healthy controls and a validation cohort limits the study’s generalizability. Nevertheless, as a pilot study, the findings are clinically significant, and a prospective study with more patients and kidney biopsy data is needed to validate these results and provide a more comprehensive understanding of GFAP’s clinical utility in AAV.

Future research on GFAP could focus on its ability to track disease progression in vasculitis, its broader utility across different forms of vasculitis, and its potential as a predictor of therapeutic response. Additionally, integrating GFAP into biomarker panels with CRP, IL-6, and ANCA could improve diagnosis and treatment monitoring in autoimmune diseases.

## 5. Conclusions

This study is the first to demonstrate that serum GFAP at diagnosis can predict cross-sectional vasculitis activity by reflecting the extent of renal involvement in patients with AAV. Additionally, the findings suggest that serum GFAP at diagnosis could potentially, albeit partially, predict progression to ESKD during follow-up in these patients. Therefore, although further validation is required, we propose that serum GFAP could serve as a valuable complementary biomarker for newly diagnosed AAV patients, particularly those with suspected renal involvement.

## Figures and Tables

**Table 1:** Characteristics of patients with AAV at diagnosis (N = 74).

Variables	Values
At diagnosis	
Demographic data	
Age (years)	63.5 (51.8−73.3)
Male sex (N, (%))	32 (43.2)
Female sex (N, (%))	42 (56.8)
AAV subtype (N, (%))	
MPA	35 (47.3)
GPA	23 (31.1)
EGPA	16 (21.6)
ANCA positivity (N, (%))	
MPO-ANCA titre	0 (0−24.5)
PR3-ANCA titre	0 (0−0)
MPO-ANCA (or P-ANCA) positive	40 (54.1)
PR3-ANCA (or C-ANCA) positive	12 (16.2)
Both ANCA positive	3 (4.1)
AAV-specific indices	
BVAS	5.0 (3.0−17.0)
FFS	0 (0−1.0)
SF-36 PCS	52.5 (35.9−67.9)
SF-36 MCS	57.0 (39.9−72.1)
VDI	3.0 (2.0−4.0)
Comorbidities (N, (%))	
Type 2 diabetes mellitus	16 (21.6)
Hypertension	22 (29.7)
Dyslipidaemia	14 (18.9)
Acute-phase reactants	
ESR (mm/h)	23.0 (7.0−85.0)
CRP (mg/L)	3.8 (0.9−28.6)
Laboratory results	
White blood cell count (/mm^3^)	7710.0 (5975.0−10,515.0)
Neutrophil count (/mm^3^)	4930.0 (3595.0−8345.0)
Lymphocyte count (/mm^3^)	1630.0 (1225.0−2145.0)
Monocyte count (/mm^3^)	480.0 (390.0−600.0)
Eosinophil count (/mm^3^)	120.0 (70.0−330.0)
Haemoglobin (g/dL)	12.5 (10.4−13.6)
Platelet count (×1000/mm^3^)	248.0 (193.0−370.5)
Blood urea nitrogen (mg/dL)	19.2 (13.7−28.7)
Serum creatinine (mg/dL)	0.8 (0.6−1.6)
Total serum protein (g/dL)	6.8 (6.5−7.3)
Serum albumin (g/dL)	4.2 (3.7−4.4)
Serum GFAP (pg/mL)	259.4 (176.4−377.4)
During follow-up	
Poor outcomes	
All-cause mortality (N, (%))	6 (8.1)
Follow-up duration based on all-cause mortality (months)	27.1 (12.0−46.4)
ESKD (N, (%))	17 (23.0)
Follow-up duration based on ESKD (months)	26.6 (8.9−46.4)
Medications (N, (%))	
Glucocorticoids	73 (98.6)
Cyclophosphamide	47 (63.5)
Rituximab	14 (18.9)
Mycophenolate mofetil	19 (25.7)
Azathioprine	45 (60.8)
Tacrolimus	7 (9.5)
Methotrexate	3 (4.1)

Values are expressed as a median (25~75 percentile) or N (%). ANCA: antineutrophil cytoplasmic antibody; AAV: ANCA-associated vasculitis; MPA: microscopic polyangiitis; GPA: granulomatosis with polyangiitis; EGPA: eosinophilic granulomatosis with polyangiitis; MPO: myeloperoxidase; PR3: proteinase 3; P: perinuclear; C: cytoplasmic; BVAS: Birmingham Vasculitis Activity Score; FFS: Five-Factor Score; SF-36: the 36-item short form survey; PCS: physical component summary; MCS: mental component summary; VDI: vasculitis damage index; ESR: erythrocyte sedimentation rate; CRP: C-reactive protein; GFAP: glial fibrillary acidic protein; ESKD: end-stage kidney disease.

## Data Availability

The dataset collected and/or analyzed in the present study are avail-able on request from the corresponding author.

## Supplementary Materials

The following supporting information can be downloaded at: https://www.mdpi.com/article/10.3390/medicina60101639/s1, Figure S1: Inductive reasoning; Figure S2: Correlation analysis of serum GFAP with creatinine clearance; Table S1: Correlation analysis of serum GFAP with continuous variables at diagnosis in patients with AAV; Table S2: Correlation analysis of serum GFAP with the sum of scores of each systemic items of BVAS at diagnosis; Table S3: Multivariable Cox proportional hazards analysis of variables at diagnosis with statistical significance in univariable Cox analysis for all-cause mortality during follow-up in AAV patients.
