# Supplementary material for: Serum Glial Fibrillary Acidic Protein Can Predict Cross-Sectional Vasculitis Activity by Reflecting Renal Involvement in Patients with Antineutrophil Cytoplasmic Antibody-Associated Vasculitis
Source: Medicina (Kaunas). 2024 Oct 7;60(10):1639. doi: 10.3390/medicina60101639 (PMC11509228; doi:10.3390/medicina60101639)
Supplement: Supplementary file 1 [file medicina-60-01639-s001.zip › SUPPLEMENTA TABLE S1(GFAP&AAV)_v02.pdf]

Supplementary Table S1. Correlation analysis of serum GFAP with continuous variables at diagnosis in patients with AAV

Variables	Correlation coefficient (r)	P-values
Age (years)	0.222	0.057
Body mass index (kg/m^2)	0.246	0.034
MPO-ANCA (or P-ANCA) titre	−0.183	0.118
PR3-ANCA (or C-ANCA) titre	−0.062	0.597
BVAS	−0.373	0.001
FFS	−0.112	0.341
SF-36 PCS	0.127	0.281
SF-36 MCS	0.103	0.384
VDI	−0.005	0.964
ESR (mm/hr)	−0.209	0.090
CRP (mg/L)	−0.320	0.006
White blood cell count (/mm^3)	−0.142	0.227
Neutrophil count (/mm^3)	−0.044	0.710
Lymphocyte count (/mm^3)	0.019	0.872
Monocyte count (/mm^3)	0.069	0.562
Eosinophil count (/mm^3)	−0.142	0.231
Haemoglobin (g/dL)	0.229	0.049
Platelet count (x1,000/mm^3)	−0.109	0.360
Fasting glucose (mg/dL)	−0.212	0.090
Total cholesterol (mg/dL)	0.093	0.452
Blood urea nitrogen (mg/dL)	0.075	0.523
Serum creatinine (mg/dL)	0.010	0.933
Total serum protein (g/dL)	−0.126	0.293
Serum albumin (g/dL)	0.232	0.049
Creatinine clearance (mL/min)	−0.14	0.233

GFAP: glial fibrillary acidic protein; ANCA: antineutrophil cytoplasmic antibody; AAV: ANCA-associated vasculitis; MPO: myeloperoxidase; PR3: proteinase 3; BVAS: the Birmingham vasculitis activity score; FFS: the five-factor score; SF36: the 36-item short form survey; PCS: physical component summary; MCS: mental component summary; VDI: vasculitis damage index; ESR: erythrocyte sedimentation rate; CRP: C-reactive protein.
