# Supplementary material for: Serum Glial Fibrillary Acidic Protein Can Predict Cross-Sectional Vasculitis Activity by Reflecting Renal Involvement in Patients with Antineutrophil Cytoplasmic Antibody-Associated Vasculitis
Source: Medicina (Kaunas). 2024 Oct 7;60(10):1639. doi: 10.3390/medicina60101639 (PMC11509228; doi:10.3390/medicina60101639)
Supplement: Supplementary file 1 [file medicina-60-01639-s001.zip › SUPPLEMENTA TABLE S2(GFAP&AAV).pdf]

Supplementary Table S2. Correlation analysis of serum GFAP with the sum of scores of each systemic items of BVAS at diagnosis

Variables	Correlation coefficient (r)	P-values
General manifestation	−0.237	0.042
Cutaneous manifestation	−0.112	0.340
Mucous and ocular manifestation	−0.138	0.241
Otorhinolaryngologic manifestation	−0.218	0.062
Pulmonary manifestation	−0.113	0.338
Cardiovascular manifestation	−0.023	0.846
Gastrointestinal manifestation	N/A	N/A
Renal manifestation	−0.335	0.003
Nervous systemic manifestation	−0.218	0.063

GFAP: glial fibrillary acidic protein; BVAS: the Birmingham vasculitis activity score.
