# Supplementary material for: Serum Glial Fibrillary Acidic Protein Can Predict Cross-Sectional Vasculitis Activity by Reflecting Renal Involvement in Patients with Antineutrophil Cytoplasmic Antibody-Associated Vasculitis
Source: Medicina (Kaunas). 2024 Oct 7;60(10):1639. doi: 10.3390/medicina60101639 (PMC11509228; doi:10.3390/medicina60101639)
Supplement: Supplementary file 1 [file medicina-60-01639-s001.zip › SUPPLEMENTA TABLE S3(GFAP&AAV).pdf]

Supplementary Table S3 Multivariable Cox proportional hazards analysis of variables at diagnosis with statistical significance in univariable Cox analysis for all-cause mortality during follow-up in AAV patients

Variables	Multivariable (SF-36 PCS ≤50.16)		
	HR	95% CI	P value
BVAS	0.951	0.839, 1.078	0.431
ESR	1.018	1.001, 1.035	0.041
CRP	0.998	0.971, 1.025	0.884
Serum creatinine	1.762	1.071, 2.898	0.026
Serum GFAP ≤194.9 pg/mL	3.208	0.855, 12.040	0.084

AAV: ANCA-associated vasculitis; ANCA: antineutrophil cytoplasmic antibody; BVAS: the Birmingham vasculitis activity score; ESR: erythrocyte sedimentation rate; CRP: C-reactive protein, GFAP: glial fibrillary acidic protein.
