# Supplementary figures and images for: Serum Glial Fibrillary Acidic Protein Can Predict Cross-Sectional Vasculitis Activity by Reflecting Renal Involvement in Patients with Antineutrophil Cytoplasmic Antibody-Associated Vasculitis
Source: Medicina (Kaunas). 2024 Oct 7;60(10):1639. doi: 10.3390/medicina60101639 (PMC11509228; doi:10.3390/medicina60101639)

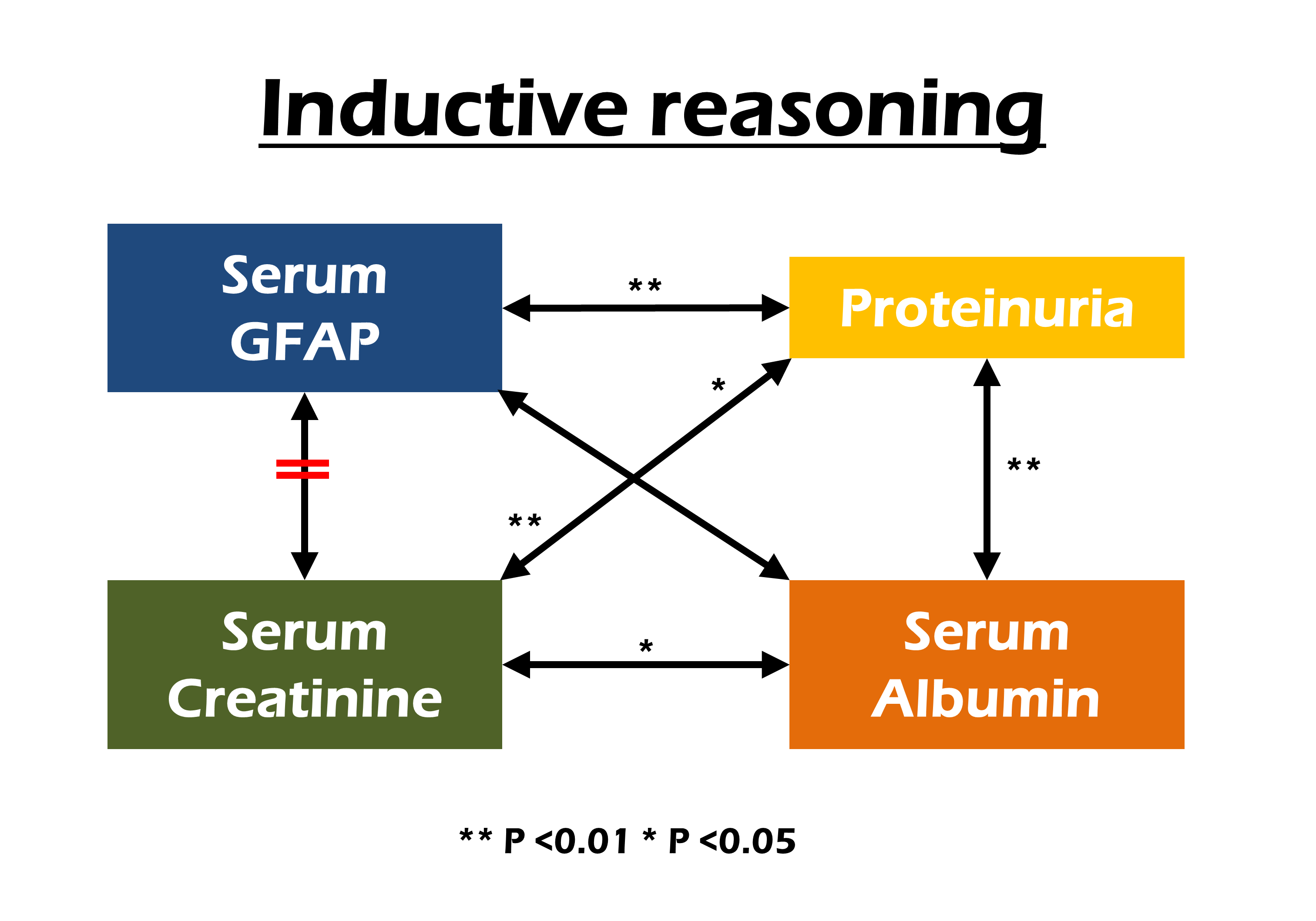

Supplement: Supplementary file 1 [file medicina-60-01639-s001.zip › SUPPLEMENTARY FIGURE S1(GFAP&AAV)(2024.6.7).tif]
